# Unilateral uterine artery embolization and Bakri tamponade balloon insertion in the treatment of acute puerperal uterine inversion: a case report

**DOI:** 10.1186/s13256-022-03419-2

**Published:** 2022-05-14

**Authors:** Wataru Isono, Akira Tsuchiya, Asuka Okamura, Michiko Honda, Ako Saito, Hiroko Tsuchiya, Reiko Matsuyama, Akihisa Fujimoto, Osamu Nishii

**Affiliations:** Department of Obstetrics and Gynaecology, University Hospital Mizonokuchi, Teikyo University School of Medicine, 5-1-1 Futago, Takatsu-ku, Kawasaki, Kanagawa 213-8507 Japan

**Keywords:** Uterine inversion, Uterine artery embolization, Bakri balloon tamponade, Uterine atony, Disseminated intravascular coagulation, Case report

## Abstract

**Background:**

Acute puerperal uterine inversion is rare but may cause massive postpartum blood loss due to uterine atony. Therefore, these patients must be diagnosed, and uterine replacement must be performed as soon as possible. However, in some cases, active bleeding due to uterine atony becomes uncontrollable, even though the uterine inversion itself is treated. In these cases, additional treatments, including surgical procedures, are needed.

**Case presentation:**

A 41-year-old Japanese woman, gravida 1, para 0, was hospitalized for labor induction at 40 weeks and 3 days of gestational age. She had a vacuum-assisted delivery after 3 days of oxytocin administration, but acute uterine inversion occurred. Although replacement of the inverted uterus was successful by manual repositioning and Bakri balloon tamponade insertion, massive postpartum hemorrhage caused by uterine atony became uncontrollable. In this situation, since disseminated intravascular coagulation had developed, we used uterine artery embolization to stop the bleeding. After detecting the pseudo-aneurysmal sac and tortuous vessels of the right uterine artery, transcatheter right-sided uterine artery embolization was performed. Thirteen days after uterine artery embolization, she was discharged with no complications.

**Conclusions:**

In cases of disseminated intravascular coagulation caused by massive postpartum bleeding, uterine artery embolization may often be selected. In our case, since we performed angiography to detect the main bleeding site, the hemorrhage could be stopped with unilateral uterine artery embolization alone, without hysterectomy.

## Background

Acute puerperal uterine inversion is rare, occurring in only approximately 1 in 3500–20,000 vaginal deliveries [1, 2]. However, since it causes massive postpartum blood loss due to uterine atony, prompt diagnosis and management are needed for replacement of the inverted uterus and control of the hemorrhage. When the treatments are performed early, the patient recovers with relatively less invasive management, such as manual replacement and Bakri postpartum balloon insertion into the repaired uterine cavity [3–6]. On the other hand, if postpartum bleeding becomes uncontrollable, various invasive interventions, including blood transfusion and surgical approaches, may be needed. In these cases, the management of hemorrhage after replacement tends to become more difficult than the replacement itself. Among these procedures, hysterectomy might be required in some cases, and very occasionally, maternal death can occur [1]. However, to preserve future fertility, avoidance of this management approach is desirable, and transcatheter arterial embolization of the uterine artery is one option to consider for managing postpartum hemorrhage [7].

Therefore, we describe a case in which we selected unilateral uterine artery embolization (UAE) without hysterectomy to manage a massive hemorrhage and coagulopathy that occurred after successful replacement of the uterine inversion itself.

## Case presentation

**Fig. 1:**
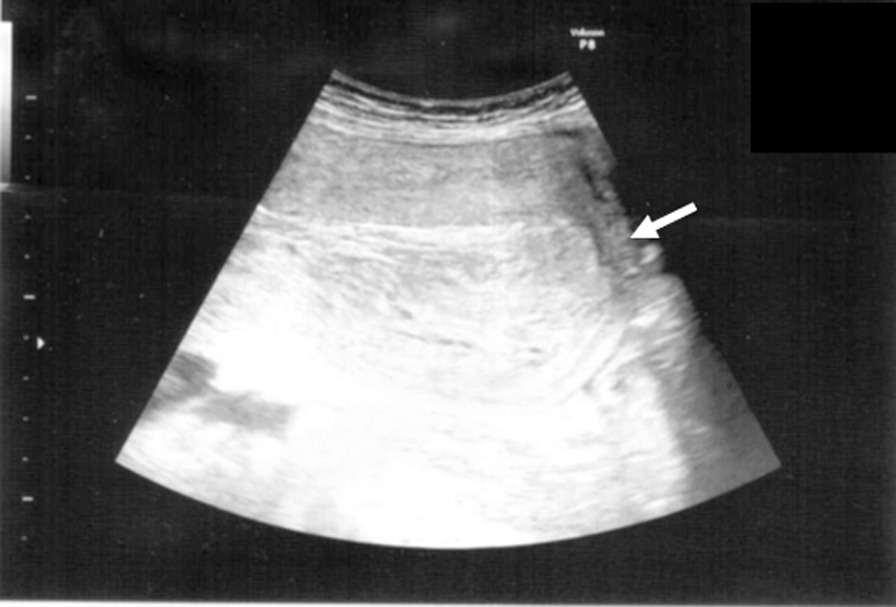
The uterine fundus (arrow) passes through the cervical ring, though this is not clear in the image

A 41-year-old Japanese female homemaker, gravida 1, para 0, with no remarkable medical, surgical, family, social, environmental, smoking, or alcohol history, was referred to our hospital at 9 gestational weeks after *in vitro* fertilization. During the gestational period, she was diagnosed with subchorionic hemorrhage in an emergency examination at 15 gestational weeks and hospitalized for treatment of a threatened premature delivery with oral ritodrine hydrochloride at 33–34 gestational weeks. At 40 weeks and 3 days of gestational age, she was hospitalized for labor induction, and after 3 days of oxytocin administration, she had a live birth; the neonate was female, weighed 3140 g, had Apgar scores of 9 and 10, and was delivered via a combination of midline episiotomy, vacuum extraction, and the Kristeller maneuver. However, while removing the placenta, a large mass, including the uterus and placenta, emerged, and uterine inversion was diagnosed by vaginal examination and abdominal ultrasonography (Fig. 1).

**Fig. 2:**
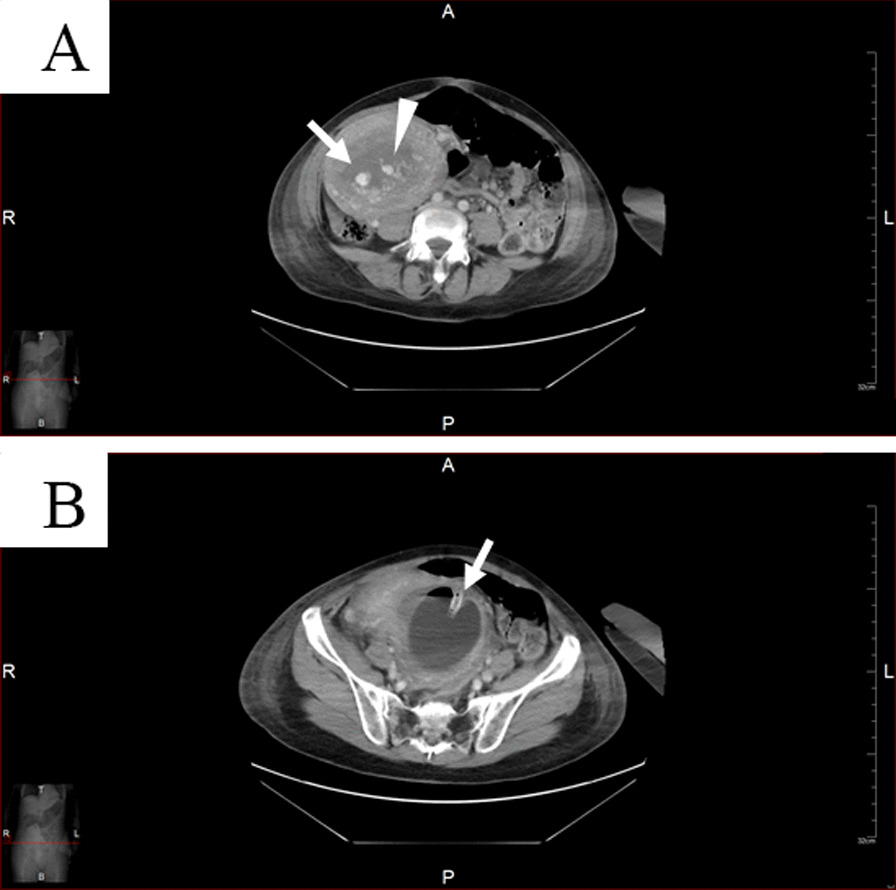
**A** The uterus was enlarged and deviated to the right. A pseudo-aneurysm-like cyst (arrow) and dilated blood vessel (arrowhead) were detected in a branch of the right uterine artery. **B** The uterine cavity was remarkably enlarged over the size of the Bakri balloon (arrow). Enlarged tortuous vessels were detected, and continuous active extravasations were suspected

**Fig. 3:**
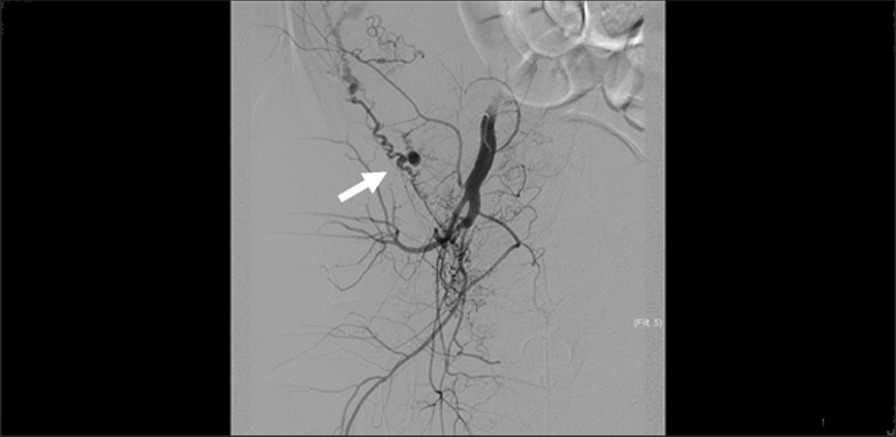
A pseudoaneurysm-like cyst (arrow) arising from a branch of the right uterine artery and enlarged tortuous vessels were detected

**Fig. 4:**
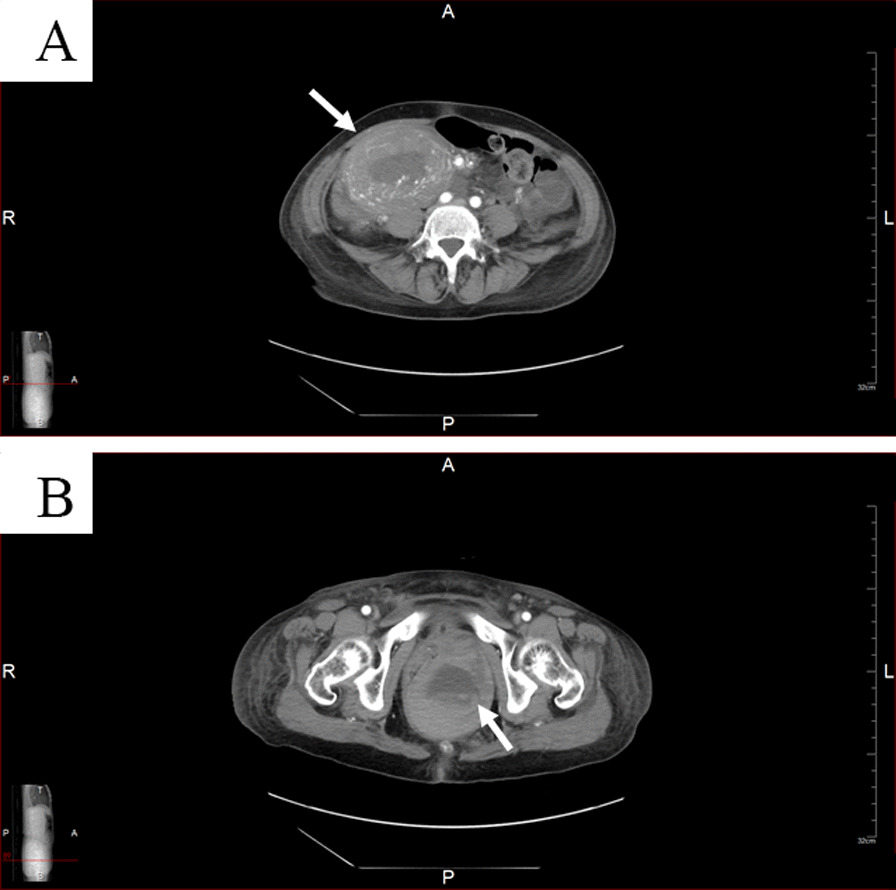
**A** Hematoma in the uterine cavity (arrow) remained, but active bleeding was not detected. **B** A large hematoma existed in the left vaginal wall (arrow), but active extravasations were not detected

Massive bleeding from the stripped uterine cavity continued, and she presented with a shock index of over 2, a pulse rate of 134 beats per minute (bpm), and a blood pressure of 41/19 mmHg. The patient had a decreased level of consciousness, with a Japan Coma Scale score of 10 [8], namely this patient could be easily aroused by being spoken to, and she had cold extremities. Since laboratory tests revealed minimal hemoglobin, platelet, and fibrinogen levels of 6.6 g/dl, 5.4 × 10^4^/μl, and 102 mg/dl, respectively, we started rapid blood transfusion and systemic management in the intensive care unit (ICU). The patient’s liver and renal function were not abnormal; her serum alanine aminotransferase, aspartate aminotransferase, and creatinine levels were 15 units/l, 15 units/l, and 0.48 mg/dl, respectively. Uterine replacement was performed with intravenous nitroglycerine (100 mg), and vaginal bleeding was controlled by Bakri balloon insertion into the recovered uterine cavity, with an iodoform gauze inside the vagina and with sutures of the perineal and vaginal lacerations. However, disseminated intravascular coagulation (DIC) had developed, and the Japanese obstetrical DIC score was 15 points [9], with a platelet count of 5.4 × 10^4^/μl, a fibrinogen level of 102 mg/dl, a prothrombin time (PT) of 16.8 seconds, an activated partial thromboplastin time (APTT) of 47.5 seconds, a fibrin degradation product (FDP) level of 97.8 μg/ml, and d-dimer of 42.0 μg/ml. Then, we performed contrast-enhanced computed tomography (CT; Fig. 2) and uterine artery angiography to detect another source of bleeding related to uterine atony. After these procedures, we decided to perform UAE instead of surgical management to preserve future fertility and avoid the risk of perioperative hemorrhaging secondary to DIC. Finally, transcatheter right-sided UAE was performed to control the pseudo-aneurysmal sac and tortuous vessels of the right uterine artery (Fig. 3). On the day of delivery, we rapidly performed blood transfusion, Bakri balloon tamponade, and UAE to control the massive bleeding. Two days later, her condition stabilized, and she was transferred from the ICU to the general ward. We detected a hematoma that was 10 cm in diameter in the left vaginal wall on the same day, so surgical removal with an indwelling catheter in place was performed under general anesthesia on the following day after confirming no active bleeding by contrast-enhanced CT (Fig. 4) and explaining the procedure to the patient and her husband. The administration of antibiotics was also needed for 18 days, including 5 days of intravenous administration of cefmetazole (2 g/day) and 13 days of oral cefcapene pivoxil (300 mg/day); however, this catheter was removed, and the vaginal wall hematoma had almost disappeared before discharge.

Overall, we measured approximately 4500 ml of blood loss and performed transfusion of 44 units of red blood cell concentrate (approximately 6160 ml), 30 units of fresh frozen plasma (approximately 3600 ml), and 20 units of platelet concentrate (approximately 400 ml), yet she was able to be discharged 13 days after delivery. Iron agents were needed for 22 days, including 3 days of intravenous saccharated ferric oxide (40 mg/day) and 19 days of oral sodium ferrous citrate (100 mg/day). Placental histopathological examination revealed placenta accrete, including increased syncytial knot, necrosis, and smooth muscle adhesion. In the outpatient department, we detected a thick endometrium approximately 4 months after UAE without difficulty, and no abnormality was detected for over 1 year.

## Discussion and conclusions

We recently encountered a patient with acute puerperal uterine inversion. She underwent combined treatment with Bakri tamponade balloon insertion, for uterine replacement, unilateral UAE, for bleeding control, and DIC without surgical interventions. Since we could preserve her fertility by performing unilateral UAE, a minimally invasive procedure, we reported this case with consideration of reviews of past reports [3–6, 10, 11].

Uterine inversion is due to the collapse of the uterine fundus into the uterine cavity. The diagnosis itself may be relatively easy because the uterine fundus can be palpated by vaginal examination and because its abdominal ultrasound findings are typical. However, this disease is a life-threatening obstetrical emergency because the condition can result in significant, persistent blood loss due to subsequent uterine atony and uterine muscle tissue necrosis due to disruption of uterine arterial blood flow [10, 11]. When uterine inversion is recognized, immediate replacement must be performed, and close follow-up is required. If prompt diagnosis and appropriate treatment are performed, in most cases, patients can recover with fewer invasive approaches, such as Bakri balloon tamponade insertion [3–6]. We also tried to control the bleeding by this intervention first. However, in some cases, when unavoidable, we must choose to perform surgical management, including hysterectomy, to control fatal bleeding.

In our case, since the development of DIC was detected before choosing surgical management, UAE was performed to control postpartum bleeding. As a result, we could avoid a hysterectomy and preserve future fertility. Additionally, before UAE was started, the dominant bleeding site was detected by contrast-enhanced CT and uterine artery angiography, and we could control the bleeding by only right-sided embolization. This minimal use of embolization may have further significance in terms of the negative impact of UAE on fertility [12–14]. A large hematoma was detected in the left vaginal wall the day after UAE due to severe DIC. This was possibly caused by the right-sided UAE, but the effectiveness of UAE for controlling postpartum hemorrhage is recognized [15, 16]. Our management reveals one option for treating uterine inversion.

In summary, we needed to perform UAE to control acute bleeding under DIC. However, since we performed angiography to detect the main bleeding site, hemorrhage could be stopped with unilateral UAE alone, without hysterectomy. To the best of our knowledge, this case is the first report in which acute uterine inversion accompanied by DIC could be managed with unilateral UAE. Although further investigation is needed, this technique may be less invasive and, therefore, better preserve future fertility.

## Data Availability

The authors agree to make all data of this study freely available.

## Abbreviations

APTT: Activated partial thromboplastin time
bpm: Beats per minute
CT: Computed tomography
DIC: Disseminated intravascular coagulation
FDP: Fibrin degradation product
ICU: Intensive care unit
PT: Prothrombin time
UAE: Uterine artery embolization

## References

[1] Coad SL, Dahlgren LS, Hutcheon JA. Risks and consequences of puerperal uterine inversion in the United States, 2004 through 2013. Am J Obstet Gynecol. 2017;217(3):377–377. 10.1016/j.ajog.2017.05.018 28522320

[2] Witteveen T, van Stralen G, Zwart J, van Roosmalen J. Puerperal uterine inversion in the Netherlands: a nationwide cohort study. Acta Obstet Gynecol Scand. 2013;92(3):334–7. 10.1111/j.1600-0412.2012.01514.x 22881867

[3] Kaya B, Tuten A, Celik H, Misirlioglu M, Unal O. Non-invasive management of acute recurrent puerperal uterine inversion with Bakri postpartum balloon. Arch Gynecol Obstet. 2014;289(3):695–6. 10.1007/s00404-013-2993-7 23974277

[4] Thiam M, Niang MM, Gueye L, Sarr FR, Dieme ME, Cisse ML. Puerperal uterine inversion managed by the uterine balloon tamponade. Pan Afr Med J. 2015;22:331. 10.11604/pamj.2015.22.331.7823 PMC4769814 26977239

[5] Vivanti AJ, Furet E, Nizard J. Successful use of a Bakri tamponade balloon in the treatment of puerperal uterine inversion during caesarean section. J Gynecol Obstet Hum Reprod. 2017;46(1):101–2. 10.1016/j.jgyn.2016.03.009 28403951

[6] Matsubara S. Combination of an intrauterine balloon and the “holding the cervix” technique for hemostasis of postpartum hemorrhage and for prophylaxis of acute recurrent uterine inversion. Acta Obstet Gynecol Scand. 2014;93(3):314. 10.1111/aogs.12282 24127992

[7] Tanahashi Y, Goshima S, Kondo H, *et al*. Transcatheter arterial embolization for primary postpartum hemorrhage: predictive factors of need for embolic material conversion of gelatin sponge particles to *N*-butyl cyanoacrylate. Cardiovasc Intervent Radiol. 2017;40(2):236–44. 10.1007/s00270-016-1496-x 27812783

[8] Ono K, Wada K, Takahara T, Shirotani T. Indications for computed tomography in patients with mild head injury. Neurol Med Chir (Tokyo). 2007;47(7):291–7. 10.2176/nmc.47.291 17652914

[9] Morikawa M, Matsunaga S, Makino S, *et al*. Effect of hypofibrinogenemia on obstetrical disseminated intravascular coagulation in Japan in 2018: a multicenter retrospective cohort study. Int J Hematol. 2021;114:18–34. 10.1007/s12185-021-03119-5 33710511

[10] Wendel MP, Shnaekel KL, Magann EF. Uterine inversion: a review of a life-threatening obstetrical emergency. Obstet Gynecol Surv. 2018;73(7):411–7. 10.1097/OGX.0000000000000580 30062382

[11] Katsura D, Moritani S, Tsuji S, *et al*. Uncontrollable uterine atony after replacement of uterine inversion managed by hysterectomy: a case report. J Med Case Rep. 2020;14(1):181. 10.1186/s13256-020-02528-0 PMC7542967 33028411

[12] Karlsen K, Hrobjartsson A, Korsholm M, Mogensen O, Humaidan P, Ravn P. Fertility after uterine artery embolization of fibroids: a systematic review. Arch Gynecol Obstet. 2018;297(1):13–25. 10.1007/s00404-017-4566-7 29052017

[13] Keung JJ, Spies JB, Caridi TM. Uterine artery embolization: a review of current concepts. Best Pract Res Clin Obstet Gynaecol. 2018;46:66–73. 10.1016/j.bpobgyn.2017.09.003 29128204

[14] Jitsumori M, Matsuzaki S, Endo M, *et al*. Obstetric outcomes of pregnancy after uterine artery embolization. Int J Womens Health. 2020;12:151–8. 10.2147/IJWH.S236443 PMC7064279 32184677

[15] Ohmaru-Nakanishi T, Kuramoto K, Maehara M, Takeuchi R, Oishi H, Ueoka Y. Complications and reproductive outcome after uterine artery embolization for retained products of conception. J Obstet Gynaecol Res. 2019;45(10):2007–14. 10.1111/jog.14067 31338948

[16] Wang CY, Pan HH, Chang CC, Lin CK. Outcomes of hypogastric artery ligation and transcatheter uterine artery embolization in women with postpartum hemorrhage. Taiwan J Obstet Gynecol. 2019;58(1):72–6. 10.1016/j.tjog.2018.11.014 30638485

